# Dapagliflozin in patients with heart failure with mildly reduced and preserved ejection fraction treated with a mineralocorticoid receptor antagonist or sacubitril/valsartan

**DOI:** 10.1002/ejhf.2722

**Published:** 2022-11-07

**Authors:** Mingming Yang, Jawad H. Butt, Toru Kondo, Karola S. Jering, Kieran F. Docherty, Pardeep S. Jhund, Rudolf A. de Boer, Brian L. Claggett, Akshay S. Desai, Adrian F. Hernandez, Silvio E. Inzucchi, Mikhail N. Kosiborod, Carolyn S.P. Lam, Anna Maria Langkilde, Felipe A. Martinez, Magnus Petersson, Sanjiv J. Shah, Muthiah Vaduganathan, Ulrica Wilderäng, Scott D. Solomon, John J.V. McMurray

**Affiliations:** ^1^ British Heart Foundation Cardiovascular Research Centre University of Glasgow Glasgow UK; ^2^ Department of Cardiology, Zhongda Hospital, School of Medicine Southeast University Nanjing China; ^3^ Department of Cardiology Copenhagen University Copenhagen Denmark; ^4^ Department of Cardiology Nagoya University Graduate School of Medicine Nagoya Japan; ^5^ Cardiovascular Division Brigham and Women's Hospital Boston MA USA; ^6^ Erasmus Medical Center Rotterdam The Netherlands; ^7^ Duke University Medical Center Durham NC USA; ^8^ Yale School of Medicine New Haven CT USA; ^9^ Saint Luke's Mid America Heart Institute University of Missouri Kansas City MS USA; ^10^ National Heart Center Singapore and Duke–National University of Singapore Singapore; ^11^ Late‐Stage Development, Cardiovascular, Renal and Metabolism BioPharmaceuticals Research and Development Gothenburg Sweden; ^12^ National University of Cordoba Cordoba Colombia; ^13^ Northwestern University Feinberg School of Medicine Chicago IL USA

**Keywords:** Mineralocorticoid receptor antagonist, Aldosterone, Neprilysin, Sacubitril/valsartan, Sodium–glucose cotransporter 2, Heart failure, Ejection fraction

## Abstract

**Aims:**

The effects of adding a sodium–glucose cotransporter 2 (SGLT2) inhibitor to a mineralocorticoid receptor antagonist (MRA) or an angiotensin receptor–neprilysin inhibitor (ARNI) in patients with heart failure (HF) and mildly reduced ejection fraction (HFmrEF) and preserved ejection fraction (HFpEF) are uncertain, even though the use of all three drugs is recommended in recent guidelines.

**Methods and results:**

The efficacy and safety of dapagliflozin added to background MRA or ARNI therapy was examined in patients with HFmrEF/HFpEF enrolled in the DELIVER trial. The primary outcome was the composite of worsening HF or cardiovascular death. Of 6263 patients, 2667 (42.6%) were treated with an MRA and 301 (4.8%) with an ARNI at baseline. Patients taking either were younger, more often men and had lower systolic blood pressure and ejection fraction; they were also more likely to have prior HF hospitalization. The benefit of dapagliflozin was similar whether patients were receiving these therapies. The hazard ratio for the effect of dapagliflozin compared to placebo on the primary outcome was 0.86 (95% confidence interval [CI] 0.74–1.01) for MRA non‐users versus 0.76 (95% CI 0.64–0.91) for MRA users (*p*_interaction_ = 0.30). The corresponding values for ARNI non‐users and users were 0.82 (95% CI 0.73–0.92) and 0.74 (95% CI 0.45–1.22), respectively (*p*_interaction_ = 0.75). None of the adverse events examined was more common with dapagliflozin compared to placebo overall or in the MRA and ARNI subgroups.

**Conclusions:**

The efficacy and safety of dapagliflozin were similar, regardless of background treatment with an MRA or ARNI. SGLT2 inhibitors may be added to other treatments recommended in recent guidelines for HFmrEF/HFpEF.

## Graphical abstract

Effects of dapagliflozin in patients taking or not taking a mineralocorticoid receptor antagonist (MRA) and an angiotensin receptor–neprilysin inhibitor (ARNI). CI, confidence interval; CV, cardiovascular; HF, heart failure; HFmrEF, heart failure with mildly reduced ejection fraction; HFpEF, heart failure with preserved ejection fraction; hosp., hospitalization.

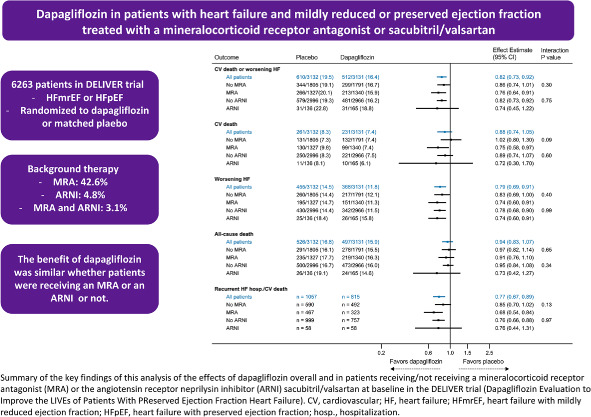

## Introduction

Recent guidelines have recommended considering the use of a mineralocorticoid receptor antagonist (MRA) and the angiotensin receptor–neprilysin inhibitor (ARNI) sacubitril/valsartan in patients with heart failure and mildly reduced ejection fraction (HFmrEF).^1^, ^2^ US guidelines also recommend the use of these drugs in selected patients with heart failure and preserved ejection fraction (HFpEF), particularly among patients with left ventricular ejection fraction (LVEF) on the lower end of this spectrum.^2^ However, the strongest recommendation is for sodium–glucose cotransporter 2 (SGLT2) inhibitors and the evidence supporting the use of SGLT2 inhibitors has been strengthened by the results of the DELIVER (Dapagliflozin Evaluation to Improve the LIVEs of Patients With PReserved Ejection Fraction Heart Failure) trial.^2^, ^3^, ^4^, ^5^, ^6^ In DELIVER, 6263 patients with heart failure and an ejection fraction >40% were randomized to dapagliflozin or placebo and followed for a median of 2.3 years. The primary outcome, time to first worsening heart failure event or cardiovascular death, was reduced significantly by dapagliflozin (hazard ratio [HR] 0.82; 95% confidence interval [CI] 0.73–0.92; *p* < 0.001).^4^

Key questions for clinicians raised by these new guidelines are about the efficacy and tolerability/safety of combinations of these different therapies for patients with HFmrEF/HFpEF.^7^, ^8^, ^9^ Although the three classes of treatment are believed to work through distinct mechanisms and should have additive benefits, the evidence that this is the case is limited. Indeed, in the EMPEROR‐Preserved trial, the effect of empagliflozin on first and recurrent heart failure hospitalizations was more pronounced in MRA non‐users (HR 0.60, 95% CI 0.47–0.77) than in MRA users (HR 0.90, 95% CI 0.68–1.19; *p*_interaction_ = 0.038) although the interaction for the primary composite outcome including cardiovascular death was not significant.^7^

While combination therapy appears well tolerated in patients with heart failure and reduced ejection fraction (HFrEF), patients with HFmrEF/HFpEF are considerably older and more comorbid, with worse kidney function.^10^, ^11^, ^12^ Therefore, more information on the tolerability of combination therapy is important to inform clinical practice.

In this pre‐specified analysis of DELIVER, we carried out a detailed evaluation of the efficacy and safety of dapagliflozin in patients who were and were not receiving background therapy with an MRA, an ARNI, or both. DELIVER is the largest trial to date in heart failure patients with an ejection fraction >40% and had a larger proportion of patients treated with an MRA than in any prior trial in such patients.^4^, ^13^, ^14^

## Methods

### Trial design and study population

The design and results of DELIVER have been reported previously.^4^, ^13^, ^14^ Briefly, DELIVER was a global, randomized, double‐blind, placebo‐controlled, event‐driven trial, which accessed the efficacy and safety of dapagliflozin 10 mg daily with a matching placebo in patients with heart failure and mildly reduced and preserved LVEF. The ethics committee at each site approved the protocol, and all patients provided written informed consent. The trial is registered with ClinicalTrials.gov (NCT03619213).

Both ambulatory and hospitalized heart failure patients were eligible if they were >40 years of age and had at least intermittent use of a diuretic agent, New York Heart Association (NYHA) functional class II–IV, LVEF >40%, evidence of structural heart disease, and N‐terminal pro‐B‐type natriuretic peptide (NT‐proBNP) >300 pg/ml (>600 pg/ml if atrial fibrillation/flutter on the electrocardiogram at enrolment). Key exclusion criteria were type 1 diabetes, estimated glomerular filtration rate (eGFR) <25 ml/min/1.73 m^2^, and systolic blood pressure <95 mmHg. A complete list of exclusion criteria is provided in the design paper.^13^

The use of drugs and devices at baseline was captured by the electronic case report form.

### Study outcomes

The primary outcome in the trial was a composite of cardiovascular death or worsening heart failure, which included unplanned heart failure hospitalization or urgent heart failure visit requiring intravenous therapy, analysed as the time‐to‐first event. Secondary outcomes included the total number of heart failure events (first and repeat heart failure hospitalizations or an urgent heart failure visit) and cardiovascular death; cardiovascular death; all‐cause death; and change in the Kansas City Cardiomyopathy Questionnaire (KCCQ) total symptom score (TSS) from baseline to 8 months. In the present study, we also examined the change in systolic blood pressure, body weight, creatinine from baseline to 1 year, and eGFR from baseline to 2 years. Among patients who were not treated with MRA or ARNI at baseline, we examined the initiation of these drugs. Finally, among individuals who were treated with MRA or ARNI at baseline, we examined the discontinuation of these drugs.

The pre‐specified safety analyses included serious adverse events, adverse events leading to discontinuation of randomized treatment, and selected adverse events, including volume depletion, renal adverse events, amputation, major hypoglycaemia, and diabetic ketoacidosis. Safety analyses were only performed in patients who received at least one dose of dapagliflozin or placebo (a total of 10 randomized patients were excluded from these analyses).

### Statistical analysis

Baseline characteristics are presented as means with standard deviations (SD), medians with interquartile ranges and frequencies with percentages. Differences in baseline characteristics were tested with the Wilcoxon test and two‐sample Student's *t*‐test for non‐normally and normally distributed continuous variables, respectively, and the chi‐square test for binary or categorical variables. Cox proportional hazards models, stratified according to diabetes status and adjusted for treatment group assignment, were used to analyse time‐to‐event data, and HRs with 95% CI were reported. Semiparametric proportional rates models, also stratified according to diabetes status and adjusted for treatment group assignment, were used to examine total (first and recurrent) events,^15^ and rate ratios (RRs) with 95% CI were reported. The changes in KCCQ‐TSS from baseline to 8 months following randomization were analysed using mixed‐effect models for repeated measurements, with adjustment for baseline value, visit (months 1, 4, and 8), treatment‐group assignment, and interaction between treatment and visit. The changes in other longitudinal measures (i.e. systolic blood pressure, body weight, and creatinine) from baseline to 1 year were analysed using mixed‐effect models for repeated measurements, with adjustment for baseline value, visit, treatment‐group assignment, and interaction between treatment and visit for the mixed‐effect models, and the least‐squares mean differences with 95% CI were reported. Finally, changes in eGFR from baseline to subsequent study visits were plotted in the MRA subgroup, and the slope was reported as per ml/min/1.73 m^2^ per year at the first visit (at 1 month) and thereafter until 24 months. These analyses were not done in the ARNI subgroup because of the small number of patients.

All analyses were conducted using Stata version 17.0 (Stata Corp, College Station, TX, USA). A *p*‐value <0.05 was considered statistically significant.

## Results

Among the 6263 participants, 2667 (42.6%) patients were receiving an MRA and 301 (4.8%) an ARNI at baseline (*Table* *1*). Of the 2667 patients taking an MRA, 197 (7.4%) were also receiving an ARNI and of the 301 patients taking an ARNI at baseline, 197 (65.4%) were prescribed an MRA as well (*Table* *1* and online supplementary *Table* *S1*).

**Table 1:** Baseline characteristics of patients according to the use of mineralocorticoid receptor antagonist and angiotensin receptor–neprilysin inhibitor

	Not on MRA (*n* = 3596)	On MRA (*n* = 2667)	*p*‐value	Not on ARNI (*n* = 5962)	On ARNI (*n* = 301)	*p*‐value
**Demographic characteristics**						
Age, years	72.8 ± 9.0	70.2 ± 10.0	<0.001	71.8 ± 9.4	68.7 ± 11.7	<0.001
Sex			0.024			<0.001
Female	1621 (45.1)	1126 (42.2)		2654 (44.5)	93 (30.9)	
Male	1975 (54.9)	1541 (57.8)		3308 (55.5)	208 (69.1)	
Region			<0.001			<0.001
North America	622 (17.3)	229 (8.6)		789 (13.2)	62 (20.6)	
Latin America	670 (18.6)	511 (19.2)		1150 (19.3)	31 (10.3)	
Europe and Saudi Arabia	1707 (47.5)	1298 (48.7)		2920 (49.0)	85 (28.2)	
Asia	597 (16.6)	629 (23.6)		1103 (18.5)	123 (40.9)	
Race			<0.001			<0.001
White	2636 (73.3)	1803 (67.6)		4280 (71.8)	159 (52.8)	
Black or African American	104 (2.9)	55 (2.1)		152 (2.5)	7 (2.3)	
Asian	637 (17.7)	637 (23.9)		1149 (19.3)	125 (41.5)	
Other	219 (6.1)	172 (6.4)		381 (6.4)	10 (3.3)	
**Physiological measurements**						
SBP, mmHg	130.1 ± 15.2	125.7 ± 15.1	<0.001	128.7 ± 15.2	119.8 ± 15.6	<0.001
DBP, mmHg	74.0 ± 10.6	73.9 ± 10.0	0.78	74.0 ± 10.3	71.6 ± 10.4	<0.001
Baseline pulse, bpm	70.9 ± 11.9	72.2 ± 11.5	<0.001	71.5 ± 11.8	70.5 ± 10.7	0.13
BMI, kg/m^2^	30.0 ± 6.1	29.6 ± 6.1	0.003	29.9 ± 6.1	28.6 ± 5.8	<0.001
**Past medical history**						
Hypertension	3253 (90.5)	2300 (86.2)	<0.001	5324 (89.3)	229 (76.1)	<0.001
Atrial fibrillation	1998 (55.6)	1467 (55.0)	0.66	3323 (55.7)	142 (47.2)	0.004
Myocardial infarction	841 (23.4)	798 (29.9)	<0.001	1550 (26.0)	89 (29.6)	0.17
Diabetes mellitus	1691 (47.0)	1115 (41.8)	<0.001	2686 (45.1)	120 (39.9)	0.078
Chronic obstructive pulmonary disease	419 (11.7)	273 (10.2)	0.077	665 (11.2)	27 (9.0)	0.24
**HF characteristics and investigations**						
Time since HF diagnosis			0.023			0.064
0–3 months	338 (9.4)	230 (8.6)		541 (9.1)	27 (9.0)	
>3–6 months	359 (10.0)	233 (8.7)		572 (9.6)	20 (6.6)	
>6–12 months	441 (12.3)	401 (15.0)		812 (13.6)	30 (10.0)	
>1–2 years	574 (16.0)	421 (15.8)		945 (15.9)	50 (16.6)	
>2–5 years	916 (25.5)	653 (24.5)		1475 (24.8)	94 (31.2)	
>5 years	965 (26.9)	727 (27.3)		1612 (27.1)	80 (26.6)	
Enrollment during or within 30 days after hospitalization for HF	312 (8.7)	342 (12.8)	<0.001	625 (10.5)	29 (9.6)	0.64
Previous hospitalization for HF	1339 (37.2)	1200 (45.0)	<0.001	2392 (40.1)	147 (48.8)	0.003
NYHA functional class			0.003			0.19
I/II	2756 (76.6)	1958 (73.4)		4497 (75.4)	217 (72.1)	
III/IV	840 (23.4)	709 (26.6)		1465 (24.6)	84 (27.9)	
Quality of life scores						
KCCQ clinical summary score	68.2 ± 20.5	68.5 ± 21.0	0.54	67.9 ± 20.7	76.1 ± 18.3	<0.001
KCCQ total summary score	66.9 ± 20.2	66.3 ± 20.3	0.26	66.4 ± 20.3	72.3 ± 17.9	<0.001
KCCQ total symptom score	70.0 ± 22.0	70.1 ± 22.4	0.79	69.6 ± 22.2	78.4 ± 19.3	<0.001
ECG findings and NT‐proBNP						
Atrial fibrillation/flutter	1475 (41.0)	1169 (43.8)	0.027	2542 (42.7)	102 (33.9)	0.003
NT‐proBNP, pg/ml	989 (604–1714)	1050 (647–1802)	0.001	1012 (622–1742)	974 (628–1892)	0.73
Atrial fibrillation/flutter on ECG	1430 (968–2173)	1381 (952–2295)	0.68	1394 (964–2206)	1557 (895–2326)	0.64
No atrial fibrillation/flutter on ECG	688 (455–1216)	748.5 (480–1400)	0.001	710 (466–1270)	796 (526–1408)	0.034
LVEF and other laboratory investigations						
LVEF, %	55.3 ± 8.6	52.7 ± 8.7	<0.001	54.5 ± 8.7	48.3 ± 7.3	<0.001
≥41–49%	1014 (28.2)	1098 (41.2)		1919 (32.2)	193 (64.1)	
≥50%	2581 (71.8)	1566 (58.7)		4039 (67.7)	108 (35.9)	
Prior LVEF ≤40%	571 (15.9)	580 (21.7)	<0.001	999 (16.8)	152 (50.5)	<0.001
Creatinine, μmol/L	103.3 ± 32.7	101.4 ± 28.7	0.019	102.3 ± 31.2	105.8 ± 28.7	0.054
eGFR, ml/min/1.73 m^2^	60.2 ± 19.3	62.1 ± 18.9	<0.001	61.0 ± 19.1	61.9 ± 19.6	0.40
eGFR ≥60 ml/min/1.73 m^2^	1789 (49.7)	1403 (52.6)	0.024	3035 (50.9)	157 (52.2)	0.67
**Medication and other interventions**						
Diuretics	3456 (96.1)	2667 (100.0)	<0.001	5834 (97.9)	289 (96.0)	0.035
Loop diuretic	2847 (79.2)	1964 (73.6)	<0.001	4576 (76.8)	235 (78.1)	0.60
Furosemide equivalent dose^a^, mg/day	48.2 ± 55.5	55.2 ± 68.0	<0.001	51.3 ± 61.8	46.1 ± 42.5	0.22
Thiazide diuretic	627 (17.4)	180 (6.7)	<0.001	795 (13.3)	12 (4.0)	<0.001
Digitalis	138 (3.8)	158 (5.9)	<0.001	276 (4.6)	20 (6.6)	0.11
Beta‐blocker	2887 (80.3)	2290 (85.9)	<0.001	4924 (82.6)	253 (84.1)	0.51
ACEi	1260 (35.0)	1035 (38.8)	0.002	2292 (38.4)	3 (1.0)	<0.001
ARB	1391 (38.7)	881 (33.0)	<0.001	2262 (37.9)	10 (3.3)	<0.001
ARNI	104 (2.9)	197 (7.4)	<0.001	N/A	N/A	
CCB	1266 (35.2)	649 (24.3)	<0.001	2470 (41.4)	197 (65.4)	<0.001
MRA	N/A	N/A		1889 (31.7)	26 (8.6)	<0.001
Pacemaker	384 (10.7)	278 (10.4)	0.75	614 (10.3)	48 (15.9)	0.002
CRT‐P or CRT‐D	50 (1.4)	50 (1.9)	0.13	82 (1.4)	18 (6.0)	<0.001
ICD	54 (1.5)	59 (2.2)	0.037	89 (1.5)	24 (8.0)	<0.001
ICD (including CRT‐D)	76 (2.1)	92 (3.5)	0.001	131 (2.2)	37 (12.3)	<0.001

Data are presented as mean ± standard deviation, median (interquartile range) for continuous measures, and *n* (%) for categorical measures.

ACEi, angiotensin‐converting enzyme inhibitor; ARB, angiotensin receptor blocker; ARNI, angiotensin receptor–neprilysin inhibitor; BMI, body mass index; CCB, calcium channel blocker; CRT‐D cardiac resynchronization therapy‐defibrillator; CRT‐P cardiac resynchronization therapy‐pacemaker; DBP, diastolic blood pressure; ECG, electrocardiogram; eGFR, estimated glomerular filtration rate; HF, heart failure; ICD, implantable cardioverter defibrillator; KCCQ, Kansas City Cardiomyopathy Questionnaire; LVEF, left ventricular ejection fraction; MRA, mineralocorticoid receptor antagonist; N/A, not applicable; NT‐proBNP, N‐terminal pro B‐type natriuretic peptide; NYHA, New York Heart Association; SBP, systolic blood pressure.

^a^ Dose was calculated as equivalent of furosemide, presented as mean ± standard deviation.

**Table 2:** Clinical outcomes by randomized treatment in patients with and without mineralocorticoid receptor antagonist

	NotonMRA		OnMRA		*p*_interaction_
	Placebo (*n* = 1805)	Dapagliflozin (*n* = 1791)	Placebo (*n* = 1327)	Dapagliflozin (*n* = 1340)	
CV death or worsening HF^a^					0.30
No. of events (%)	344 (19.1)	299 (16.7)	266 (20.1)	213 (15.9)	
Rate per 100 patient‐years (95% CI)	9.2 (8.3–10.3)	7.9 (7.1–8.9)	10.1 (9.0–11.4)	7.7 (6.8–8.8)	
HR (95% CI)	0.86 (0.74–1.01)		0.76 (0.64–0.91)		
Worsening HF^a^					0.40
No. of events (%)	260 (14.4)	217 (12.1)	195 (14.7)	151 (11.3)	
Rate per 100 patient‐years (95% CI)	7.0 (6.2–7.9)	5.7 (5.0–6.6)	7.4 (6.4–8.5)	5.5 (4.7–6.4)	
HR (95% CI)	0.83 (0.69–1.00)		0.74 (0.60–0.91)		
HF hospitalization^a^					0.28
No. of events (%)	236 (13.1)	195 (10.9)	182 (13.7)	134 (10.0)	
Rate per 100 patient‐years (95% CI)	6.3 (5.5–7.1)	5.1 (4.5–5.9)	6.9 (5.9–7.9)	4.8 (4.1–5.7)	
HR (95% CI)	0.82 (0.68–1.00)		0.71 (0.56–0.88)		
CV death^a^					0.09
No. of events (%)	131 (7.3)	132 (7.4)	130 (9.8)	99 (7.4)	
Rate per 100 patient‐years (95% CI)	3.2 (2.7–3.8)	3.3 (2.8–3.9)	4.5 (3.8–5.4)	3.4 (2.8–4.1)	
HR (95% CI)	1.02 (0.80–1.30)		0.75 (0.58–0.97)		
All‐cause death^a^					0.65
No. of events (%)	291 (16.1)	278 (15.5)	235 (17.7)	219 (16.3)	
Rate per 100 patient‐years (95% CI)	7.2 (6.4–8.0)	6.9 (6.1–7.8)	8.2 (7.2–9.3)	7.5 (6.6–8.6)	
HR (95% CI)	0.97 (0.82–1.14)		0.91 (0.76–1.10)		
Recurrent HF events/CV death^a^					0.13
No. of events	590	492	467	323	
Rate per 100 patient‐years (95% CI)	14.6 (12.9–16.7)	12.3 (10.8–14.1)	16.3 (14.1–19.1)	11.1 (9.5–13.1)	
RR (95% CI)	0.85 (0.70–1.02)		0.68 (0.54–0.84)		
KCCQ total symptom score					0.93
Change from baseline to 8 months (95% CI)	5.7 (4.8–6.7)	8.1 (7.1–9.0)	5.3 (4.3–6.4)	7.8 (6.7–8.8)	
Placebo‐corrected change at 8 months (95% CI)	2.3 (1.0–3.6)		2.4 (0.9–3.9)		

CI, confidence interval; CV, cardiovascular; HF, heart failure; HR, hazard ratio; KCCQ, Kansas City Cardiomyopathy Questionnaire; MRA, mineralocorticoid receptor antagonist; RR, rate ratio.

^a^ Stratified by diabetes status and adjusted for treatment assignment.

**Table 3:** Clinical outcomes by randomized treatment in patients with and without angiotensin receptor–neprilysin inhibitor

	NotonARNI		OnARNI		*p*_interaction_
	Placebo (*n* = 2996)	Dapagliflozin (*n* = 2966)	Placebo (*n* = 136)	Dapagliflozin (*n* = 165)	
CV death or worsening HF^a^					0.75
No. of events (%)	579 (19.3)	481 (16.2)	31 (22.8)	31 (18.8)	
Rate per 100 patient‐years (95% CI)	9.5 (8.7–10.3)	7.7 (7.1–8.4)	13.5 (9.5–19.2)	10.5 (7.4–14.9)	
HR (95% CI)	0.82 (0.73–0.92)		0.74 (0.45–1.22)		
Worsening HF^a^					0.99
No. of events (%)	430 (14.4)	342 (11.5)	25 (18.4)	26 (15.8)	
Rate per 100 patient‐years (95% CI)	7.0 (6.4–7.7)	5.5 (4.9–6.1)	10.9 (7.4–16.1)	8.8 (6.0–12.9)	
HR (95% CI)	0.78 (0.68–0.90)		0.77 (0.44–1.33)		
HF hospitalization^a^					0.78
No. of events (%)	394 (13.2)	303 (10.2)	24 (17.7)	26 (15.8)	
Rate per 100 patient‐years (95% CI)	6.4 (5.8–7.0)	4.8 (4.3–5.4)	10.4 (7.0–15.5)	8.8 (6.0–12.9)	
HR (95% CI)	076 (0.65–0.88)		0.79 (0.45–1.39)		
CV death^a^					0.60
No. of events (%)	250 (8.3)	221 (7.5)	11 (8.1)	10 (6.1)	
Rate per 100 patient‐years (95% CI)	3.8 (3.3–4.2)	3.3 (2.9–3.8)	4.3 (2.4–7.8)	3.1 (1.7–5.8)	
HR (95% CI)	0.89 (0.74–1.07)		0.72 (0.30–1.70)		
All‐cause death^a^					0.34
No. of events (%)	500 (16.7)	473 (16.0)	26 (19.1)	24 (14.6)	
Rate per 100 patient‐years (95% CI)	7.5 (6.9–8.2)	7.1 (6.5–7.8)	10.1 (6.9–14.8)	7.5 (5.0–11.2)	
HR (95% CI)	0.95 (0.84–1.08)		0.73 (0.42–1.27)		
Recurrent HF events/CV death^a^					0.97
No. of events	999	757	58	58	
Rate per 100 patient‐years (95% CI)	15.0 (13.6–16.7)	11.5 (10.3–12.8)	22.8 (15.4–35.3)	18.3 (12.3–28.4)	
RR (95% CI)	0.76 (0.66–0.88)		0.76 (0.44–1.31)		
KCCQ total symptom score					0.32
Change from baseline to 8 months (95% CI)	5.7 (5.0–6.4)	8.2 (7.5–8.9)	2.9 (−0.3 to 6.1)	2.8 (0.0 to 5.7)	
Placebo‐corrected change at 8 months (95% CI)	2.5 (1.5–3.5)		−0.1 (−4.4–4.2)		

ARNI, angiotensin receptor neprilysin inhibitor; CI, confidence interval; CV, cardiovascular; HF, heart failure; HR, hazard ratio; KCCQ, Kansas City Cardiomyopathy Questionnaire; RR, rate ratio.

^a^ Stratified by diabetes status and adjusted for treatment assignment.

**Figure 1:**
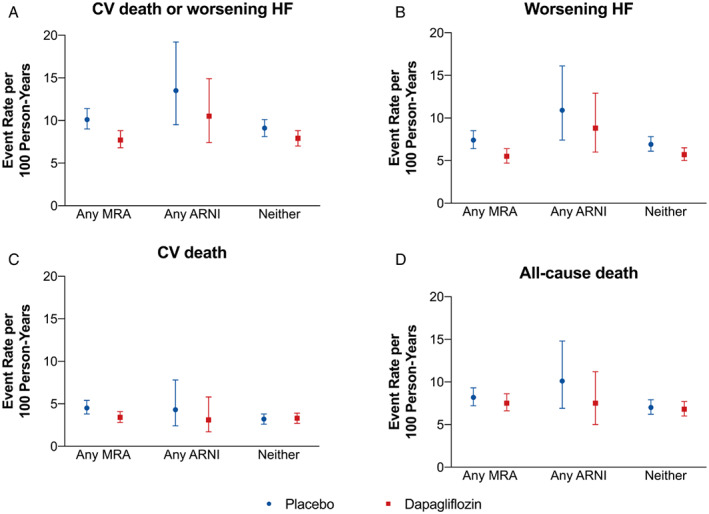
Event rates in patients taking a mineralocorticoid receptor antagonist (MRA) or angiotensin receptor–neprilysin inhibitor (ARNI) or neither drug at baseline. CV, cardiovascular; HF, heart failure.

### Baseline characteristics of patients according to baseline treatment

#### Baseline treatment with a mineralocorticoid receptor antagonist

##### Demographics, physiologic measures and medical history

Compared to patients not taking an MRA, those treated with an MRA were younger, less likely to have been enrolled in North America, and had a lower mean systolic blood pressure and body mass index but higher eGFR. Patients treated with an MRA were also less likely to have a history of hypertension and type 2 diabetes.

##### Heart failure history, characteristics and treatment

Compared to patients not taking an MRA, those treated with an MRA were more likely to have a history of heart failure hospitalization and had a lower mean LVEF but similar KCCQ scores. They were also more likely to have a prior LVEF ≤40% and to have been enrolled during or shortly after hospitalization for heart failure.

Participants treated with an MRA were more likely to be receiving a beta‐blocker and have a defibrillating device. Patients receiving an MRA at baseline were more likely to be taking a loop than a thiazide diuretic and were on a higher mean dose of loop diuretic compared to people not receiving an MRA at baseline (*Table* *1*). The mean (± SD) daily MRA dose was 29.7 ± 16.6 mg in the placebo group and 29.5 ± 16.4 in the dapagliflozin group (*p* = 0.80).

Within the MRA subgroup, baseline characteristics were well balanced among patients assigned to dapagliflozin versus placebo (online supplementary *Table S* *2*).

#### Baseline treatment with an angiotensin receptor–neprilysin inhibitor

##### Demographics, physiologic measures and medical history

The differences between patients treated with an ARNI and not treated with an ARNI were similar to those observed for MRA therapy, although patients treated with an ARNI were more likely to have been enrolled in North America (and patients treated with an MRA less likely to be recruited in that region). Patients treated with an ARNI were also less likely to have atrial fibrillation than those not treated with an ARNI, a difference not observed for MRA therapy.

##### Heart failure history, characteristics and treatment

Compared to patients not taking an ARNI, those treated with an ARNI were more likely to have a history of heart failure hospitalization and had a lower mean LVEF, similar to what was observed for MRA therapy. However, KCCQ scores were better in patients receiving an ARNI compared to those not (this difference was not seen with MRA therapy). Patients treated with an ARNI at baseline were more likely to have a prior LVEF ≤40% and to have been enrolled during or shortly after hospitalization for heart failure. Participants treated with an ARNI were more likely to have a defibrillating device and cardiac resynchronization therapy.

Within the subgroup defined by ARNI use, baseline characteristics were well balanced among patients assigned to dapagliflozin versus placebo (online supplementary *Table S* *2*).

#### Efficacy outcomes and effects of dapagliflozin according to baseline treatment

Overall, crude event rates were similar in MRA users and non‐users. However, patients taking ARNI had numerically higher event rates than those not receiving an ARNI, although the differences were not statistically significant (*Figure* *1*, *Tables* 2, 3, online supplementary *Table S* *2*).

##### Effects of dapagliflozin according to background mineralocorticoid receptor antagonist treatment

The benefit of dapagliflozin on the primary outcome was consistent in patients taking an MRA or not taking an MRA: the HR (95%CI) for dapagliflozin compared with placebo was 0.86 (0.74–1.01) in participants not taking an MRA compared with 0.76 (0.64–0.91) in patients taking an MRA (*p*_interaction_ = 0.30) (*Table* *2* and *Graphical Abstract*). The benefit of dapagliflozin added to an MRA was consistent in patients with an eGFR <60 and ≥60 ml/min/1.73 m^2^ (online supplementary *Figure* *S1*). A consistent picture was also seen for other outcomes (*Table* *2*, online supplementary *Table* *S2*, and *Graphical Abstract*). In particular (in light of the EMPEROR‐Preserved findings described in the Introduction), the rate ratio for total (first and repeat) heart failure events and cardiovascular death was 0.85 (0.70–1.02) in patients not treated with an MRA at baseline and 0.68 (0.54–0.84) in participants receiving an MRA (*p*_interaction_ = 0.13).

**Figure 2:**
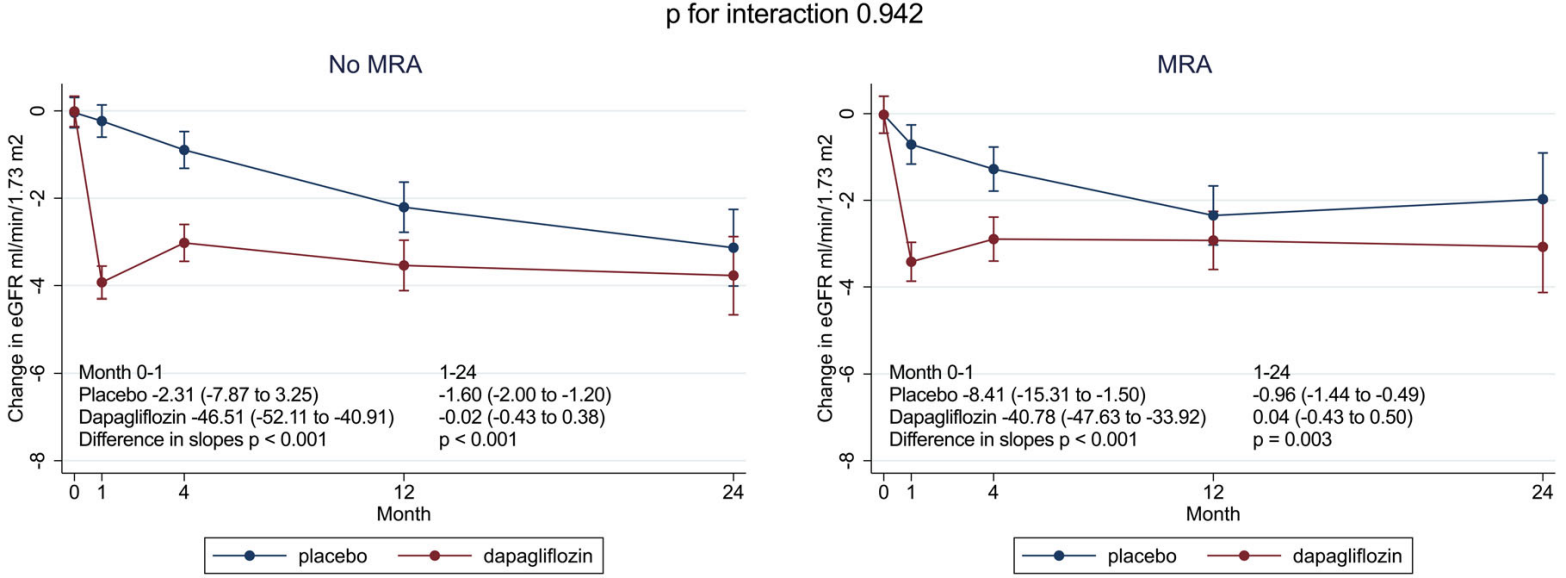
Change in estimated glomerular filtration rate (eGFR) during follow‐up by randomized treatment in patients with and without mineralocorticoid receptor antagonist (MRA). Changes in eGFR slope are shown as per ml/min/1.73 m^2^.

In addition, there was a similar increase (improvement) in KCCQ‐TSS at 8 months in patients treated or not treated with an MRA at baseline (*Table* *2*). The initial decrease (‘dip’) in eGFR with dapagliflozin was similar in users and non‐users of an MRA at baseline (*Figure* *2*). The rate of decline in eGFR after 1 month (‘chronic eGFR slope’, months 1–24) was slowed by dapagliflozin, compared with placebo, to a similar extent in patients treated and not treated with an MRA at baseline (*Figure* *2*).

##### Effects of dapagliflozin according to background angiotensin receptor–neprilysin inhibitor treatment

The benefit of dapagliflozin on the primary outcome was similar in patients taking an ARNI or not taking an ARNI: the HR (95%CI) for dapagliflozin compared with placebo was 0.82 (0.73–0.92) in participants not taking an ARNI compared with 0.74 (0.45–1.22) in patients taking an ARNI (*p*_interaction_ = 0.75) (*Table* *3* and *Graphical Abstract*). The benefit of dapagliflozin added to an ARNI was consistent in patients with an eGFR <60 and ≥60 ml/min/1.73 m^2^ (online supplementary *Figure* *S1*). A similar picture was also seen for other outcomes (*Table* *3*, online supplementary *Table S* *2* and *Graphical Abstract*). There were too few patients in the ARNI subgroup to calculate eGFR slopes.

Although only 197 (3.1%) patients were prescribed both an MRA and ARNI at baseline, the effect of dapagliflozin on the primary endpoint in these participants (HR 0.86, 95% CI 0.45–1.64) was consistent with what was observed in the trial overall (HR 0.82, 95% CI 0.73–0.92) (online supplementary *Table S* *2*).

While not pre‐specified, we also examined the effects of dapagliflozin according to background treatment with other therapies (diuretic, angiotensin‐converting enzyme inhibitor/angiotensin receptor blocker or beta‐blocker) and found a consistent benefit across these additional subgroups (online supplementary *Table S* *3*).

#### Effects of dapagliflozin on physiologic measures and safety outcomes according to baseline treatment with a mineralocorticoid receptor antagonist or angiotensin receptor–neprilysin inhibitor

##### Systolic blood pressure, serum creatinine, and weight

The placebo‐corrected change in systolic blood pressure from baseline to 1 year with dapagliflozin did not differ significantly according to baseline MRA treatment: −1.29 mmHg in patients not taking an MRA versus −0.64 mmHg in those receiving an MRA (*p*_interaction_ = 0.42). The findings were similar for the ARNI subgroup: −1.11 mmHg in those not taking an ARNI compared with 0.79 mmHg in patients treated with an ARNI at baseline (*p*_interaction_ = 0.27) (*Table* *4*). The overall picture was similar for change in creatinine and weight.

**Table 4:** Changes in repeated measurements by randomized treatment in patients stratified by the use of mineralocorticoid receptor antagonist and angiotensin receptor–neprilysin inhibitor

	Not on MRA		On MRA		*p*_interaction_	NotonARNI		OnARNI		*p*_interaction_
	Placebo (*n* = 1805)	Dapagliflozin (*n* = 1791)	Placebo (*n* = 1327)	Dapagliflozin (*n* = 1340)		Placebo (*n* = 2996)	Dapagliflozin (*n* = 2966)	Placebo (*n* = 136)	Dapagliflozin (*n* = 165)	
Systolic blood pressure, mmHg					0.42					0.27
Change from baseline to 1 year	1.14 ± 0.39	−0.15 ± 0.39	0.48 ± 0.43	−0.16 ± 0.43		0.91 ± 0.30	−0.20 ± 0.30	−0.06 ± 1.34	0.72 ± 1.26	
Difference^a^	−1.29 (−2.37 to −0.21); *p* = 0.019		−0.64 (−1.83 to 0.55); *p* = 0.314			−1.11 (−1.93 to −0.29); *p* = 0.008		0.79 (−2.82 to 4.40); *p* = 0.669		
Creatinine, mg/dl					0.15					0.23
Change from baseline to 1 year	0.06 ± 0.01	0.09 ± 0.01	0.05 ± 0.01	0.06 ± 0.01		0.05 ± 0.01	0.08 ± 0.01	0.04 ± 0.02	0.01 ± 0.02	
Difference^a^	0.03 (0.01 to 0.05); *p* = 0.009		0.01 (−0.02 to 0.03); *p* = 0.571			0.02 (0.01 to 0.04); *p* = 0.008		−0.03 (−0.09 to 0.03); *p* = 0.370		
Weight, kg					0.84					0.47
Change from baseline to 1 year	−0.15 ± 0.14	−1.25 ± 0.14	0.11 ± 0.14	−0.93 ± 0.14		−0.04 ± 0.10	−1.08 ± 0.10	−0.24 ± 0.49	−1.67 ± 0.46	
Difference^a^	−1.10 (−1.48 to −0.71); *p* < 0.001		−1.04 (−1.43 to −0.65); *p* < 0.001			−1.04 (−1.32 to −0.76); *p* < 0.001		−1.43 (−2.74 to −0.12); *p* = 0.032		

ARNI, angiotensin receptor–neprilysin inhibitor; MRA, mineralocorticoid receptor antagonist.

^a^ Difference between dapagliflozin and placebo at 1 year with 95% confidence interval. Stratified by diabetes status and adjusted for treatment assignment.

Discontinuation of dapagliflozin, compared to placebo, was not greater overall or among patients receiving background MRA or ARNI therapy. None of the adverse events examined differed between dapagliflozin and placebo overall, or according to background MRA or ARNI therapy (*Table* *5*).

**Table 5:** Discontinuation and safety outcomes by randomized treatment in patients stratified by the use of mineralocorticoid receptor antagonist and angiotensin receptor–neprilysin inhibitor

	Not on MRA		On MRA		*p*_interaction_	Not on ARNI		On ARNI		*p*_interaction_
	Placebo (*n* = 1805)	Dapagliflozin (*n* = 1791)	Placebo (*n* = 1327)	Dapagliflozin (*n* = 1340)		Placebo (*n* = 2996)	Dapagliflozin (*n* = 2966)	Placebo (*n* = 136)	Dapagliflozin (*n* = 165)	
Any discontinuation	262 (14.5)	286 (16.0)	180 (13.6)	158 (11.8)	0.07	420 (14.0)	420 (14.2)	22 (16.2)	24 (14.6)	0.68
AE leading to treatment discontinuation (DAE)	109 (6.1)	119 (6.7)	72 (5.4)	64 (4.8)	0.29	170 (5.7)	176 (5.9)	11 (8.1)	7 (4.2)	0.14
Volume depletion SAE or DAE	25 (1.4)	26 (1.5)	12 (0.9)	23 (1.7)	0.18	33 (1.1)	47 (1.6)	4 (2.9)	2 (1.2)	0.14
Renal SAE or DAE	53 (2.9)	55 (3.1)	38 (2.9)	29 (2.2)	0.29	83 (2.8)	81 (2.7)	8 (5.9)	3 (1.8)	0.07
Amputation	16 (0.9)	15 (0.8)	10 (0.8)	4 (0.3)	0.20	25 (0.8)	19 (0.6)	1 (0.7)	0 (0.0)	N/A
Major hypoglycaemia	4 (0.2)	7 (0.4)	3 (0.2)	1 (0.1)	0.17	7 (0.2)	8 (0.3)	0 (0.0)	0 (0.0)	N/A
Any definite or probable diabetic ketoacidosis	0 (0.0)	2 (0.1)	0 (0.0)	0 (0.0)	N/A	0 (0.0)	2 (0.1)	0 (0.0)	0 (0.0)	N/A
Hyperkalaemia SAE	2 (0.1)	10 (0.6)	2 (0.2)	1 (0.1)	0.09	4 (0.1)	11 (0.4)	0 (0.0)	0 (0.0)	N/A

Values are *n* (%) unless otherwise stated. Safety events were reported whether patients were on or off treatment. Ten randomized patients were excluded from the safety analysis, analyses were carried out in patients who had undergone randomization and received at least one dose of the randomized treatment.

AE, adverse event; ARNI, angiotensin receptor–neprilysin inhibitor; MRA, mineralocorticoid receptor antagonist; N/A, not applicable; SAE, serious adverse event.

#### Changes in concomitant therapy according to randomized therapy

##### Mineralocorticoid receptor antagonist therapy

**Figure 3:**
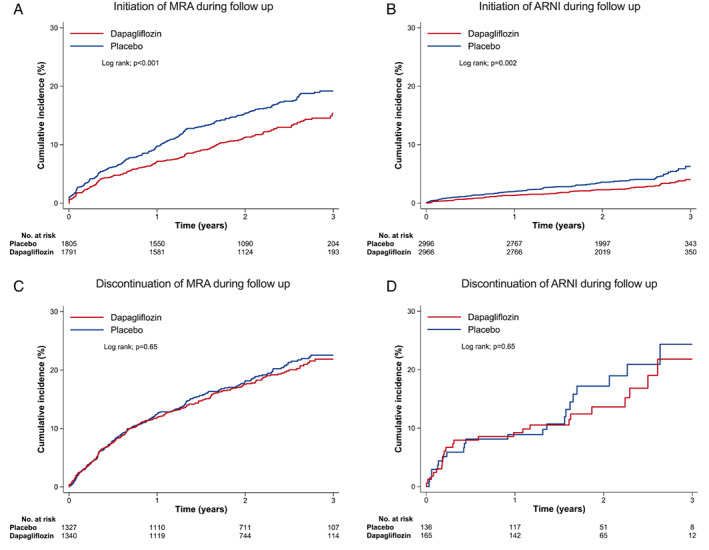
Initiation and discontinuation of a mineralocorticoid receptor antagonist (MRA) or angiotensin receptor–neprilysin inhibitor (ARNI) after randomization according to randomized treatment group.

Among patients who were not on an MRA at baseline and randomly assigned to dapagliflozin, 217/1791 patients (12.1%) started treatment with an MRA compared with 295/1805 patients (16.3%) assigned to placebo, giving a HR of 0.73 (95% CI 0.61–0.87) (*Figure* *3A*).

Among patients receiving an MRA at baseline, 243/1340 patients (18.1%) randomly assigned to dapagliflozin discontinued this treatment, compared to 251/1327 patients (18.9%) assigned to placebo stopping an MRA, giving a HR of 0.96 (95% CI 0.81–1.15) (*Figure* *3C*).

##### Sacubitril/valsartan

Among patients who were not on an ARNI at baseline and randomly assigned to dapagliflozin, 80/2966 patients (2.7%) started treatment with sacubitril/valsartan compared with 125/2996 patients (4.2%) assigned to placebo, giving a HR of 0.64 (95% CI 0.48–0.85) (*Figure* *3B*).

Regarding discontinuation of baseline treatment with an ARNI, this occurred in 24/165 patients (14.6%) assigned to dapagliflozin, and in 22/136 patients (16.2%) assigned to placebo, giving a HR of 0.85 (95% CI 0.47–1.53) (*Figure* *3D*).

## Discussion

In this pre‐specified analysis of DELIVER, we examined the efficacy and safety of dapagliflozin used in combination with an MRA or an ARNI. We found that the benefits of dapagliflozin were consistent, irrespective of background therapy with these two agents with no statistically significant interactions (in contrast to what was observed in EMPEROR‐Preserved with an MRA).^7^ Regarding tolerability and safety, again there was no evidence that background treatment with either an MRA or an ARNI modified the tolerability of dapagliflozin. These findings are of clinical importance, given that guidelines recommend considering the use of both MRA and ARNI in selected patients with mildly reduced and preserved ejection fraction.

Although it is believed that SGLT2 inhibitors and MRAs work through distinct mechanisms, these possibly could overlap to some extent for example, through initiating or facilitating diuresis. However, two large trials in patients with HFrEF showed clear benefits of adding an SGLT2 inhibitor to baseline MRA therapy, with no suggestion that background MRA treatment modified the beneficial effects or safety profile of SGLT2 inhibition.^10^, ^12^ It was therefore surprising that the EMPEROR‐Preserved investigators reported an interaction between MRA therapy and the effects of empagliflozin in patients with HFmrEF/HFpEF whereby the main benefit of empagliflozin, a reduction in heart failure hospitalization, appeared to be much more pronounced in MRA non‐users,

and greatly attenuated in MRA users.^7^ The EMPEROR‐Preserved authors reported that MRA users in their study may have been more congested and speculated that this may have influenced the observed differences in the effect of empagliflozin between MRA users and non‐users. MRA users in DELIVER may also have been more congested as a larger proportion were recruited during or shortly after hospitalization and their NT‐proBNP level was higher than in MRA non‐users. However, in DELIVER we did not observe any interaction between baseline MRA use and the effects of dapagliflozin. If anything, there was a tendency to numerically greater benefits in participants receiving an MRA at baseline, a trend also observed in both large SGLT2 inhibitor HFrEF trials.^10^, ^12^ Thus, we believe that it is more likely the interaction found in EMPEROR‐Preserved was probably due to the play of chance.

A major barrier (both perceived and real) to MRA use is renal dysfunction, and chronic kidney disease is common in patients with HFmrEF/HFpEF.^16^, ^17^ SGLT2 inhibitors also cause an initial decline (‘dip’) in eGFR and this may lead some physicians to be concerned about combining these agents in HFmrEF/HFpEF.^18^, ^19^ We observed that the initial ‘dip’ in eGFR due to dapagliflozin was similar in MRA users and non‐users in DELIVER and the benefit of dapagliflozin in slowing the long‐term rate of decline in eGFR was maintained in patients receiving background MRA therapy compared to those not receiving an MRA at baseline, as has been seen in patients with HFrEF.^10^, ^12^ We also looked specifically at the efficacy of dapagliflozin added to an MRA in patients with an eGFR <60 ml/min/1.73 m^2^ and found a consistent benefit in these patients compared to participants with an eGFR ≥60 ml/min/1.73 m^2^, again as observed in patients with HFrEF.^3^, ^4^, ^6^, ^7^, ^20^, ^21^ Overall, our data show that the cardiovascular and renal benefits of dapagliflozin are maintained in patients receiving MRA treatment and these treatments can be combined safely, as discussed below.

The effects of adding an SGLT2 inhibitor to an ARNI in patients with HFmrEF/HFpEF have not been described. However, in patients with HFrEF, the benefits of adding an SGLT2 inhibitor to sacubitril/valsartan were consistent in patients receiving and not receiving an ARNI at baseline.^11^, ^22^ Overall, only 301 participants (4.8%) were taking an ARNI at baseline in DELIVER, although the proportion was higher in patients also receiving an MRA at baseline (7.4% vs. 2.9%), as was also found in EMPEROR‐Preserved (3.8% vs. 1.3%). Approximately half of the patients treated with sacubitril/valsartan at baseline had a history of a prior LVEF ≤40% which is perhaps not surprising given that these participants were enrolled before new guidelines recommended this therapy in patients with HFmrEF/HFpEF and the main indication for an ARNI during the trial enrolment period was HFrEF.^1^, ^2^ Because of the much smaller number of patients receiving an ARNI, our findings are less robust and any inferences must be more cautious. However, the benefits of dapagliflozin once again appeared consistent in this subgroup of patients, compared to those not treated with an ARNI with no hint of an interaction. We could not analyse eGFR slopes in the ARNI subgroup because of the small number of patients taking this treatment at baseline.

Interestingly, the addition of both an MRA and an ARNI after randomization was significantly less common in the dapagliflozin group than in the placebo group. Initiation of new therapy frequently reflects the treating physician's response to a patient experiencing worsening heart failure.^23^, ^24^ Therefore, we speculate that the greater use of these two treatments in the placebo group during follow‐up likely reflects the greater risk of worsening in the placebo group compared with the dapagliflozin group.

### Limitations

A major limitation of this report is that it is about subgroups which are always underpowered and this was especially true for baseline ARNI treatment. Neither MRA nor ARNI treatment was randomized. Potassium concentration was not measured during follow‐up

and only investigator‐reported serious adverse events were available for hyperkalaemia. The patients studied were enrolled in a clinical trial with inclusion and exclusion criteria and thus were relatively selected compared with the overall population of patients with HFmrEF/HFpEF. The data relating to treatment with an ARNI may not be generalizable to all patients with HFmrEF/HFpEF as in this study half had prior HFrEF. Other limitations are the under‐representation of Black patients and the absence of serial measurements of NT‐proBNP.

## Conclusions

Among patients with HFmrEF/HFpEF, we found that the benefits and safety of dapagliflozin were similar when added to background treatment with an MRA or an ARNI. The clinical decision to initiate SGLT2 inhibitors in this patient group should not be contingent on the background use of an MRA or ARNI.

### Funding

John J.V. McMurray is supported by a British Heart Foundation Centre of Research Excellence Grant RE/18/6/34217. Mingming Yang is funded by the China Scholarship Council. The DELIVER trial was funded by AstraZeneca.

**Conflict of interest**: M.Y. has recieved a grant from AstraZeneca to attend a medical congress. J.H.B. reports advisory board honoraria from Bayer. T.K. reports speaker fees from Abbott, Ono Pharma, Otsuka Pharma, Novartis, AstraZeneca, Bristol Myers Squibb, and Abiomed. KJ has received support from the National Institutes of Health (Training Grant 5‐T32 HL007604). K.F.D. reports receiving honoraria from AstraZeneca and a research grant to his institution from Boehringer Ingelheim. P.S.J. reports other from AstraZeneca, personal fees from Novartis and Cytokinetics, and grants from Boehringer Ingelheim. R.A.d.B. has received research grants and fees (outside the submitted work) from AstraZeneca, Abbott, Boehringer Ingelheim, Cardio Pharmaceuticals Gmbh, Ionis Pharmaceuticals, Inc, Novo Nordisk, and Roche; has received speaker fees from Abbott, AstraZeneca, Bayer, Novartis, and Roche (outside the submitted work). B.L.C. has received consulting fees from Boehringer Ingelheim. A.S.D. has received grants and personal fees from AstraZeneca during the conduct of the study; personal fees from Abbott, Biofourmis, Boston Scientific, Boehringer Ingelheim, Corvidia, DalCor Pharma, Relypsa, Regeneron, and Merck; grants and personal fees from Alnylam and Novartis; and personal fees from Amgen, outside the submitted work. A.F.H. has received research support from American Regent, AstraZeneca, Boehringer Ingelheim, Merck, Novartis, and Verily; and has served as a consultant or on the Advisory Board for Amgen, AstraZeneca, Bayer, Boehringer Ingelheim, Boston Scientific, Bristol Myers Squibb, Cytokinetics, Myokardia, Merck, Novartis, and Vifor. S.E.I. has served on clinical trial committees or as a consultant to AstraZeneca, Boehringer Ingelheim, Novo Nordisk, Lexicon, Merck, Pfizer, vTv Therapeutics, Abbott, and Esperion; and has given lectures sponsored by AstraZeneca and Boehringer Ingelheim. M.N.K. has received research grant support from AstraZeneca, and Boehringer Ingelheim; has served as a consultant or on an advisory board for Alnylam, Amgen, Applied Therapeutics, AstraZeneca, Bayer, Boehringer Ingelheim, Cytokinetics, Eli Lilly, Esperion Therapeutics, Janssen, Merck (Diabetes and Cardiovascular), Novo Nordisk, Sanofi, and Vifor Pharma; has received other research support from AstraZeneca; and has received honoraria from AstraZeneca, Boehringer Ingelheim, and Novo Nordisk. C.S.P.L. is supported by a Clinician Scientist Award from the National Medical Research Council of Singapore; has received research support from AstraZeneca, Bayer, Boston Scientific, and Roche Diagnostics; has served as a consultant or on the advisory board/steering committee/executive committee for Actelion, Amgen, Applied Therapeutics, AstraZeneca, Bayer, Boehringer Ingelheim, Boston Scientific, Cytokinetics, Darma Inc, Us2.ai, Janssen Research & Development LLC, Medscape, Merck, Novartis, Novo Nordisk, Radcliffe Group Ltd, Roche Diagnostics, Sanofi, and WebMD Global LLC; and serves as the co‐founder and non‐executive director of Us2.ai. A.M.L., M.P., and U.W. are employees and shareholders of AstraZeneca. F.A.M. has received personal fees from AstraZeneca. S.J.S. has received either personal or institutional research support for DELIVER from AstraZeneca. M.V. has received research grant support or served on advisory boards for American Regent, Amgen, AstraZeneca, Bayer AG, Baxter Healthcare, Boehringer Ingelheim, Cytokinetics, Lexicon Pharmaceuticals, Novartis, Pharmacosmos, Relypsa, Roche Diagnostics, and Sanofi, speaker engagements with Novartis and Roche Diagnostics, and participates on clinical endpoint committees for studies sponsored by Galmed and Novartis. S.D.S. has received research grants from Actelion, Alnylam, Amgen, AstraZeneca, Bellerophon, Bayer, Bristol Myers Squibb, Celladon, Cytokinetics, Eidos, Gilead, GlaxoSmithKline, Ionis, Lilly, Mesoblast, MyoKardia, National Institutes of Health/NHLBI, Neurotronik, Novartis, NovoNordisk, Respicardia, Sanofi Pasteur, Theracos, US2.AI; and has consulted for Abbott, Action, Akros, Alnylam, Amgen, Arena, AstraZeneca, Bayer, Boehringer Ingelheim, Bristol Myers Squibb, Cardior, Cardurion, Corvia, Cytokinetics, Daiichi‐Sankyo, GlaxoSmithKline, Lilly, Merck, Myokardia, Novartis, Roche, Theracos, Quantum Genomics, Cardurion, Janssen, Cardiac Dimensions, Tenaya, Sanofi‐Pasteur, Dinaqor, Tremeau, CellProThera, Moderna, American Regent, and Sarepta. J.J.V.M. reports payments through Glasgow University from work on clinical trials, consulting and other activities from Alnylam, Amgen, AstraZeneca, Bayer, Boehringer Ingelheim, BMS, Cardurion, Cytokinetics, Dal‐Cor, GSK, Ionis, KBP Biosciences, Novartis, Pfizer, Theracos; personal lecture fees from Corpus, Abbott, Hikma, Sun Pharmaceuticals, Medscape/Heart.Org, Radcliffe Cardiology, Servier Director, Global Clinical Trial Partners (GCTP).

## Supporting information


**Appendix S1.** Supporting information.
