# Supplementary material for: Dapagliflozin in patients with heart failure with mildly reduced and preserved ejection fraction treated with a mineralocorticoid receptor antagonist or sacubitril/valsartan
Source: Eur J Heart Fail. 2022 Nov 7;24(12):2307–19. doi: 10.1002/ejhf.2722 (PMC11497302; doi:10.1002/ejhf.2722)

Dapagliflozin in HFrEF Patients Treated with Mineralocorticoid Receptor Antagonists and Sacubitril/Valsartan

The DELIVER Trial

Supplemental Material

| **Tables** | Page |
| --- | --- |
| Supplemental Table 1 | 2 |
| Supplemental Table 2 | 7 |
| Supplemental Table 3 | 9 |
| **Figure** |  |
| Supplemental Figure 1 | 12 |

Table 1: Baseline characteristics of patients according to the use of MRA and ARNI

|  | **None** | **On MRA** | **On ARNI** | **On MRA and ARNI** |  |
| --- | --- | --- | --- | --- | --- |
| **N** | **3492** | **2470** | **104** | **197** | ***P* value** |
| **Demographic characteristics** |  |  |  |  |  |
| Age, yr | 72.8 ± 9.0 | 70.5 ± 9.8 | 73.1±10.5 | 66.3±11.6 | <0.001 |
| Sex |  |  |  |  | <0.001 |
| Female | 1595 (45.7) | 1059 (42.9) | 26 (25.0) | 67 (34.0) |  |
| Male | 1897 (54.3) | 1411 (57.1) | 78 (75.0) | 130 (66.0) |  |
| Region |  |  |  |  | <0.001 |
| North America | 585 (16.8) | 204 (8.3) | 37 (35.6) | 25 (12.7) |  |
| Latin America | 664 (19.0) | 486 (19.7) | 6 (5.8) | 25 (12.7) |  |
| Europe and Saudi Arabia | 1675 (48.0) | 1245 (50.4) | 32 (30.8) | 53 (26.9) |  |
| Asia | 568 (16.3) | 535 (21.7) | 29 (27.9) | 94 (47.7) |  |
| Race |  |  |  |  | <0.001 |
| White | 2568 (73.5) | 1712 (69.3) | 68 (65.4) | 91 (46.2) |  |
| Black or African American | 100 (2.9) | 52 (2.1) | 4 (3.8) | 3 (1.5) |  |
| Asian | 606 (17.4) | 543 (22.0) | 31 (29.8) | 94 (47.7) |  |
| Other | 218 (6.2) | 163 (6.6) | 1 (1.0) | 9 (4.6) |  |
| Physiological measurements |  |  |  |  |  |
| SBP, mmHg | 130.3 ± 15.2 | 126.3 ± 15.0 | 121.8 ± 15.3 | 118.8 ± 15.7 | <0.001 |
| DBP, mmHg | 74.1 ± 10.6 | 74.0 ± 9.9 | 69.9 ± 9.4 | 72.4 ± 10.8 | <0.001 |
| Baseline Pulse, bpm | 71.0 ± 11.9 | 72.3 ± 11.6 | 69.9 ± 10.8 | 70.8 ± 10.7 | <0.001 |
| BMI, kg/m^2^ | 30.1 ± 6.1 | 29.7 ± 6.1 | 29.0 ± 5.9 | 28.4 ± 5.8 | <0.001 |
| Past medical history |  |  |  |  |  |
| Hypertension | 3166 (90.7) | 2158 (87.4) | 87 (83.7) | 142 (72.1) | <0.001 |
| Atrial fibrillation | 1937 (55.5) | 1386 (56.1) | 61 (58.7) | 81 (41.1) | <0.001 |
| Myocardial infarction | 813 (23.3) | 737 (29.8) | 28 (26.9) | 61 (31.0) | <0.001 |
| Diabetes Mellitus | 1649 (47.2) | 1037 (42.0) | 42 (40.4) | 78 (39.6) | <0.001 |
| Chronic obstructive pulmonary disease | 404 (11.6) | 261 (10.6) | 15 (14.4) | 12 (6.1) | 0.053 |
|  |  |  |  |  |  |
| **HF characteristics and investigations** |  |  |  |  |  |
| Time since HF diagnosis |  |  |  |  | 0.004 |
| 0-3, month | 332 (9.5) | 209 (8.5) | 6 (5.8) | 21 (10.7) |  |
| > 3-6, month | 353 (10.1) | 219 (8.9) | 6 (5.8) | 14 (7.1) |  |
| > 6-12, month | 435 (12.5) | 377 (15.3) | 6 (5.8) | 24 (12.2) |  |
| > 1-2, year | 559 (16.0) | 386 (15.6) | 15 (14.4) | 35 (17.8) |  |
| > 2-5, year | 874 (25.1) | 601 (24.4) | 42 (40.4) | 52 (26.4) |  |
| > 5, year | 936 (26.8) | 676 (27.4) | 29 (27.9) | 51 (25.9) |  |
| Enrollment during or within 30 days after hospitalization for HF | 308 (8.8) | 317 (12.8) | 4 (3.8) | 25 (12.7) | <0.001 |
| Previous hospitalization for HF | 1294 (37.1) | 1098 (44.5) | 45 (43.3) | 102 (51.8) | <0.001 |
| NYHA functional class |  |  |  |  | 0.022 |
| I/II | 2680 (76.7) | 1817 (73.6) | 76 (73.1) | 141 (71.6) |  |
| III/IV | 812 (23.3) | 653 (26.4) | 28 (26.9) | 56 (28.4) |  |
| Quality of life scores |  |  |  |  |  |
| KCCQ clinical summary score | 68.0 ± 20.5 | 67.8 ± 21.0 | 73.1 ± 18.2 | 77.7 ± 18.1 | <0.001 |
| KCCQ total summary score | 66.8 ± 20.2 | 65.7 ± 20.4 | 70.4 ± 18.0 | 73.2 ± 17.7 | <0.001 |
| KCCQ total symptom score | 69.7 ± 22.0 | 69.4 ± 22.5 | 77.2 ± 19.5 | 79.0 ± 19.3 | <0.001 |
| ECG findings and NT-proBNP |  |  |  |  |  |
| Atrial fibrillation/flutter | 1437 (41.2) | 1105 (44.7) | 38 (36.5) | 64 (32.5) | <0.001 |
| NT-proBNP, pg/ml | 987 (601-1708) | 1061 (652-1795) | 1034 (692-1970) | 951 (584-1850) | 0.005 |
| Atrial fibrillation/flutter on ECG | 1431 (972-2161) | 1367 (952-2290) | 1394 (855-2326) | 1670 (987-2373) | 0.76 |
| No atrial fibrillation/flutter on ECG | 681 (453-1201) | 748 (477-1402) | 937 (564-1677) | 759 (522-1250) | <0.001 |
| LVEF and other laboratory investigations |  |  |  |  |  |
| LVEF, % | 55.4 ± 8.6 | 53.1 ± 8.7 | 49.9 ± 7.8 | 47.5 ± 6.9 | <0.001 |
| ≥ 41-49 | 957 (27.4) | 962 (38.9) | 57 (54.8) | 136 (69.0) |  |
| ≥ 50 | 2534 (72.6) | 1505 (60.9) | 47 (45.2) | 61 (31.0) |  |
| Prior LVEF ≤ 40, % | 518 (14.8) | 481 (19.5) | 53 (51.0) | 99 (50.3) | <0.001 |
| Creatinine, μmol/L | 103.0 ± 32.8 | 101.3 ± 28.7 | 111.4 ± 29.9 | 102.9 ± 27.7 | 0.004 |
| eGFR, mL/min/1.73m^2^ | 60.3 ± 19.3 | 61.9 ± 18.8 | 57.5 ± 18.1 | 64.3 ± 20.0 | <0.001 |
| eGFR≥60, mL/min/1.73m^2^ | 1745 (50.0) | 1290 (52.2) | 44 (42.3) | 113 (57.4) | 0.025 |
|  |  |  |  |  |  |
| **Medication and other interventions** |  |  |  |  |  |
| Diuretics | 3364 (96.3) | 2470 (100.0) | 92 (88.5) | 197 (100.0) | <0.001 |
| Digitalis | 131 (3.8) | 145 (5.9) | 7 (6.7) | 13 (6.6) | <0.001 |
| Beta-blocker | 2803 (80.3) | 2121 (85.9) | 84 (80.8) | 169 (85.8) | <0.001 |
| ACEi | 1259 (36.1) | 1033 (41.8) | 1 (1.0) | 2 (1.0) | <0.001 |
| ARB | 1387 (39.7) | 875 (35.4) | 4 (3.8) | 6 (3.0) | <0.001 |
| ARNI | N/A | N/A | N/A | N/A |  |
| CCB | 1253 (35.9) | 636 (25.7) | 13 (12.5) | 13 (6.6) | <0.001 |
| Pacemaker | 364 (10.4) | 250 (10.1) | 20 (19.2) | 28 (14.2) | 0.009 |
| CRT-P or CRT-D | 44 (1.3) | 38 (1.5) | 6 (5.8) | 12 (6.1) | <0.001 |
| ICD | 40 (1.1) | 49 (2.0) | 14 (13.5) | 10 (5.1) | <0.001 |
| ICD (including CRT-D) | 59 (1.7) | 72 (2.9) | 17 (16.3) | 20 (10.2) | <0.001 |

Data are presented as mean ± SD, median (IQR) for continuous measures, and n (%) for categorical measures.

All the abbreviations are shown in Table 1.

Table 2: Clinical outcomes by randomized treatment in patients using MRA and ARNI

|  | **Neither of MRA and ARNI** | | **On MRA** | | **On ARNI** | | **Both MRA and ARNI** | |  |
| --- | --- | --- | --- | --- | --- | --- | --- | --- | --- |
|  | Placebo (n=1754) | Dapagliflozin (n=1738) | Placebo (n=1242) | Dapagliflozin (n=1228) | Placebo (n=51) | Dapagliflozin (n=53) | Placebo (n=85) | Dapagliflozin (n=112) | p Value for interaction |
| **CV death or worsening HF** |  |  |  |  |  |  |  |  | 0.50 |
| No. of events (%) | 330 (18.8) | 290 (16.7) | 249 (20.1) | 191 (15.6) | 14 (27.5) | 9 (17.0) | 17 (20.0) | 22 (19.6) |  |
| Rate per 100 patient years (95%CI) | 9.1 (8.1-10.1) | 7.9 (7.0-8.8) | 10.0 (8.8-11.3) | 7.5 (6.5-8.6) | 15.9 (9.4-26.9) | 9.1 (4.8-17.6) | 12.0 (7.5-19.4) | 11.2 (7.4-17.0) |  |
| HR (95%CI) | 0.88 (0.75-1.03) | | 0.75 (0.62-0.90) | | 0.59 (0.26-1.37) | | 0.86 (0.45-1.64) | |  |
| **Worsening HF** |  |  |  |  |  |  |  |  | 0.69 |
| No. of events (%) | 250 (14.3) | 210 (12.1) | 180 (14.5) | 132 (10.8) | 10 (19.6) | 7 (13.2) | 15 (17.7) | 19 (17.0) |  |
| Rate per 100 patient years (95%CI) | 6.9 (6.1-7.8) | 5.7 (5.0-6.5) | 7.2 (6.2-8.4) | 5.2 (4.4-6.1) | 11.4 (6.1-21.1) | 7.1 (3.4-14.9) | 10.6 (6.4-17.6) | 9.7 (6.2-15.2) |  |
| HR (95%CI) | 0.84 (0.70-1.01) | | 0.71 (0.57-0.89) | | 0.63 (0.24-1.67) | | 0.82 (0.41-1.63) | |  |
| **HF hosp.** |  |  |  |  |  |  |  |  | 0.47 |
| No. of events (%) | 226 (12.9) | 188 (10.8) | 168 (13.5) | 115 (9.4) | 10 (19.6) | 7 (13.2) | 14 (16.5) | 19 (17.0) |  |
| Rate per 100 patient years (95%CI) | 6.1 (5.4-7.0) | 5.1 (4.4-5.8) | 6.7 (5.7-7.8) | 4.5 (3.7-5.3) | 11.4 (6.1-21.1) | 7.1 (3.4-14.9) | 9.8 (5.8-16.6) | 9.7 (6.2-15.1) |  |
| HR (95%CI) | 0.83 (0.69-1.01) | | 0.67 (0.53-0.85) | | 0.63 (0.24-1.67) | | 0.87 (0.43-1.75) | |  |
| **CV death** |  |  |  |  |  |  |  |  | 0.31 |
| No. of events (%) | 125 (7.1) | 128 (7.4) | 125 (10.1) | 93 (7.6) | 6 (11.8) | 4 (7.6) | 5 (5.9) | 6 (5.4) |  |
| Rate per 100 patient years (95%CI) | 3.2 (2.6-3.8) | 3.3 (2.7-3.9) | 4.6 (3.9-5.5) | 3.5 (2.8-4.2) | 6.2 (2.8-13.7) | 3.8 (1.4-10.0) | 3.2 (1.3-7.6) | 2.8 (1.3-6.2) |  |
| HR (95%CI) | 1.04 (0.81-1.33) | | 0.75 (0.57-0.98) | | 0.61 (0.17-2.17) | | 0.94 (0.28-3.15) | |  |
| **All-cause death** |  |  |  |  |  |  |  |  | 0.82 |
| No. of events (%) | 277 (15.8) | 267 (15.4) | 223 (18.0) | 206 (16.8) | 14 (27.5) | 11 (20.8) | 12 (14.1) | 13 (11.6) |  |
| Rate per 100 patient years (95%CI) | 7.0 (6.2-7.9) | 6.8 (6.0-7.7) | 8.2 (7.2-9.4) | 7.6 (6.7-8.8) | 14.4 (8.5-24.3) | 10.3 (5.7-18.6) | 7.5 (4.3-13.2) | 6.1 (3.5-10.5) |  |
| HR (95%CI) | 0.98 (0.83-1.16) | | 0.92 (0.76-1.12) | | 0.69 (0.31-1.52) | | 0.81 (0.37-1.81) | |  |
| **Recurrent HF events/ CV death** |  |  |  |  |  |  |  |  | 0.17 |
| No. of events | 564 | 478 | 435 | 279 | 26 | 14 | 32 | 44 |  |
| Rate per 100 patient years (95%CI) | 14.3 (12.6-16.4) | 12.3 (10.7-14.1) | 16.1 (13.8-18.9) | 10.4 (8.8-12.3) | 26.8 (14.4-55.2) | 13.4 (7.0-29.1) | 20.4 (12.1-36.8) | 20.7 (12.8-35.6) |  |
| RR (95%CI) | 0.86 (0.71-1.04) | | 0.64 (0.51-0.81) | | 0.49 (0.20-1.22) | | 0.92 (0.47-1.79) | |  |
| **KCCQ - Total symptom score** |  |  |  |  |  |  |  |  | 0.51 |
| Change from baseline to 8 month (95% CI) | 5.9 (4.9-6.8) | 8.3 (7.3-9.2) | 5.5 (4.4-6.6) | 8.1 (7.0-9.2) | 0.7 (-4.6 to 6.1) | 0.0 (-5.5 to 5.6) | 4.0 (0.1-8.0) | 4.1 (0.8-7.3) |  |
| Placebo-corrected change at 8 month (95% CI) | 2.4 (1.1-3.7) | | 2.6 (1.0-4.2) | | -0.7 (-8.6 to 7.2) | | 0.0 (-5.1 to 5.2) | |  |

*Stratified by diabetes status and adjusted for treatment assignment.

ARNI, angiotensin receptor neprilysin inhibitor; CI, confidence interval; CV, cardiovascular; HF, heart failure; hosp., hospitalization; HR, hazard ratio; KCCQ, Kansas City Cardiomyopathy Questionnaire; MRA, mineralocorticoid receptor antagonist; No., number; RR, rate ratio.

Table 3a: Clinical outcomes by randomized treatment in patients receiving or not receiving a diuretic at baseline

|  | **Not on diuretics** | | **On diuretics** | | p Value for interaction |
| --- | --- | --- | --- | --- | --- |
|  | Placebo (n=70) | Dapagliflozin (n=70) | Placebo (n=3062) | Dapagliflozin (n=3061) |  |
| **CV death or worsening HF** |  |  |  |  | 0.64 |
| No. of events (%) | 14 (20.0) | 13 (18.6) | 596 (19.5) | 499 (16.3) |  |
| Rate per 100 patient-years (95%CI) | 9.9 (5.8-16.6) | 9.2 (5.3-15.8) | 9.6 (8.9-10.4) | 7.8 (7.2-8.5) |  |
| HR (95%CI) | 0.92 (0.43-1.97) | | 0.81 (0.72-0.92) | |  |

Stratified by diabetes status and adjusted for treatment assignment.

CI, confidence interval; CV, cardiovascular; HF, heart failure; HR, hazard ratio.

Table 3b: Clinical outcomes by randomized treatment in patients receiving or not receiving an ACEI or ARB at baseline

|  | **Not on ACEI and ARB** | | **On ARCE or ARB** | | p Value for interaction |
| --- | --- | --- | --- | --- | --- |
|  | Placebo (n=851) | Dapagliflozin (n=869) | Placebo (n=2281) | Dapagliflozin (n=2262) |  |
| **CV death or worsening HF** |  |  |  |  | 0.80 |
| No. of events (%) | 178 (20.9) | 149 (17.2) | 432 (18.9) | 363 (16.1) |  |
| Rate per 100 patient-years (95%CI) | 9.2 (8.3-10.3) | 7.9 (7.1-8.9) | 10.1 (9.0-11.4) | 7.7 (6.8-8.8) |  |
| HR (95%CI) | 0.80 (0.64-1.00) | | 0.83 (0.72-0.95) | |  |

Stratified by diabetes status and adjusted for treatment assignment.

ACEI, angiotensin-converting enzyme inhibitor; ARB, angiotensin receptor blocker; CI, confidence interval; CV, cardiovascular; HF, heart failure; HR, hazard ratio.

Table 3c: Clinical outcomes by randomized treatment in patients receiving or not receiving a beta-blocker at baseline

|  | **Not on beta blockers** | | **On beta blockers** | | p Value for interaction |
| --- | --- | --- | --- | --- | --- |
|  | Placebo (n=547) | Dapagliflozin (n=539) | Placebo (n=2585) | Dapagliflozin (n=2592) |  |
| **CV death or worsening HF** |  |  |  |  | 0.85 |
| No. of events (%) | 125 (22.9) | 102 (18.9) | 485 (18.8) | 410 (15.8) |  |
| Rate per 100 patient-years (95%CI) | 11.9 (10.0-14.1) | 9.4 (7.7-11.4) | 9.1 (8.4-10.0) | 7.5 (6.8-8.3) |  |
| HR (95%CI) | 0.79 (0.61-1.03) | | 0.82 (0.72-0.94) | |  |

Stratified by diabetes status and adjusted for treatment assignment.

CI, confidence interval; CV, cardiovascular; HF, heart failure; HR, hazard ratio.

Figure 1: Effect of Dapagliflozin on clinical outcomes in patients with and without MRA or ARNI according to baseline eGFR category. ARNI, angiotensin receptor neprilysin inhibitor; CV, cardiovascular; eGFR, estimated glomerular filtration rate; HF, heart failure; MRA, mineralocorticoid receptor antagonist.


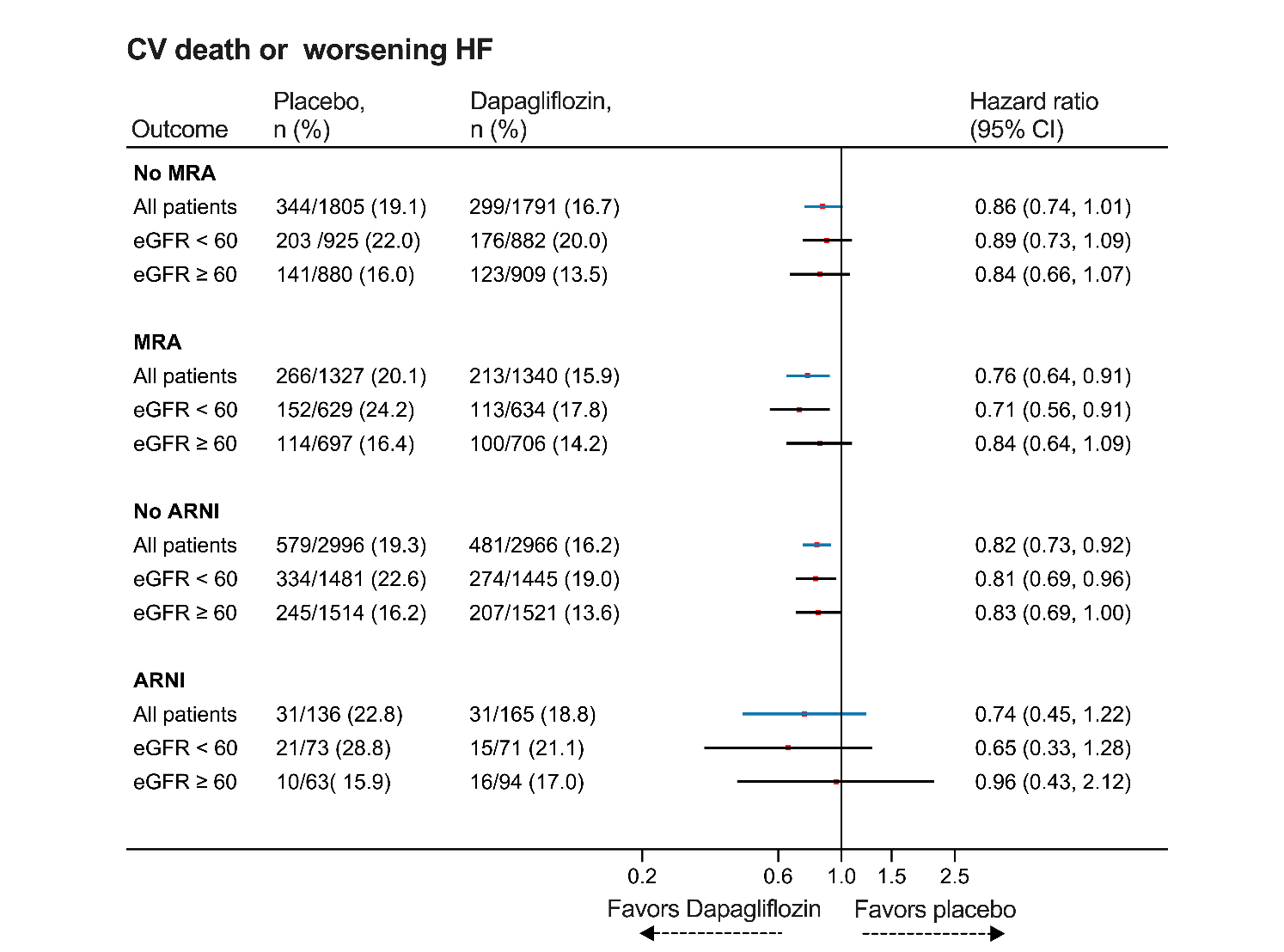

Supplement: Supplementary file 1 — Appendix S1. Supporting information. [file EJHF-24-2307-s001.docx]
